# Chorematic modeling to represent dynamics in the quinoa agroecosystems in Peru

**DOI:** 10.1371/journal.pone.0300464

**Published:** 2024-04-16

**Authors:** Francesca Fagandini Ruiz, Antonio Villanueva, Didier Bazile

**Affiliations:** 1 CIRAD, UMR TETIS, Montpellier, France; 2 TETIS, AgroParisTech, CIRAD, CNRS, INRAE, Université de Montpellier, Montpellier, France; 3 GeoMonde, Montpellier, France; 4 CIRAD, UMR SENS, Montpellier, France; 5 SENS, CIRAD, Université de Montpellier, Montpellier, France; Veracruzana University: Universidad Veracruzana, MEXICO

## Abstract

Our research occurred in the Andean region, one of the eight global centers of domestication of plant species grown for agriculture. The shores of Lake Titicaca (located between Peru and Bolivia), at 3800 meters above sea level, are recognized as the center of origin of quinoa (*Chenopodium quinoa* Willd.). In this region, complex societies have emerged, thanks to the development of water and soil management technologies. They have managed to overcome high mountain territories’ extreme and variable climatic conditions. These societies have traditionally protected and preserved quinoa as food for present and future generations through their long-standing knowledge and cultivation practices. The fieldwork occurred in the context of Andean family farming, and our study analyzes nature-society dynamics with a chorematic approach and interviews with local communities. The interest of this work is the transformation of the landscape at the scale of the mountain agroecosystem to understand better the impacts of rural development policies. Chorematic modeling was applied to two periods, before and after 1970, a pivotal year in Peru for agriculture, to show how socio-spatial dynamics in the Andean environment are changing, particularly concerning the evolution of quinoa cultivation. The results show that wild quinoa relatives’ distribution is strongly linked to the socio-spatial organization of the agroecosystem. Different species of wild quinoa relatives are maintained by villagers for their multiple foods, medicinal and cultural uses in natural areas, grazed areas, on edge, and also within cultivated fields. However, this management is changing under the pressure of global issues related to the international quinoa market, whose requirements imply reducing the presence of wild relatives in cultivated fields.

## Introduction

The Andean region is one of the eight global centers of domestication of plant species grown for agriculture [1]. The shores of Lake Titicaca (located between Peru and Bolivia), at 3800 meters above sea level, are recognized as the center of origin of *C*. *quinoa* [2]. In this region, complex societies have emerged, thanks to the development of water and soil management technologies, and they have managed to overcome the extreme and variable climatic conditions of high mountain territories [3]. These societies have traditionally protected and preserved quinoa as food for present and future generations through their long-standing knowledge and cultivation practices [4].

For centuries quinoa became an underutilized crop due to Spanish colonization processes and the introduction of new European species [5]. However, studies carried out in this region have shown that even today, a tremendous genetic diversity of quinoa is concentrated, both in cultivated varieties and in wild relatives [6–9].

In recent decades the Andean altiplano has been subject to intense pressures and uncertainties that weaken the ancestral relationship between nature and society [10–12]. The main threats to the food security of Andean subsistence agro-pastoral systems include climate change, the transformation of market systems, the implementation of non-inclusive sectoral rural development policies, the degradation of natural ecosystems, and rural migration [13–16]. The quinoa boom began around the 1980s and brought with it great upheaval [17]. The economic opportunity represented by quinoa’s commercial cultivation can impact food security, agro-biodiversity, and agroecosystem management [18, 19]. Since the quinoa boom, new institutional mechanisms have been created, new relationships between actors and societies have been developed, and new forms of production have appeared [20]. Profound changes in the relationship between society and nature accompany the economic opportunity of quinoa [21]. The local adaptation responses, strategies, and opportunities of family farming to face these transformations have consequences on the organization of the territory [10, 13, 16, 22].

Chorematic models provide valuable insights into the organization and dynamics of territories, aiding in our understanding of various issues. They can also serve as practical decision-making tools [10, 23–26]. These approaches primarily simplify regional scale elements of spatial dynamics [11]. However, incorporating the lived reality of actors at the local scale [27] and, even more challenging, at the scale of agricultural parcels [23], remains a significant endeavor. Notably, certain aspects such as pressures on environmental resources, governance, and territory administration have received limited attention within the realm of chorematic modeling [27].

The present research work was conducted specifically in the Puno region of Peru, one of the central quinoa-producing regions in the world [5]. The cultivated varieties of quinoa and its wild relatives show a spatial distribution along a north-south climatic gradient and a differentiation into agroecological zones related to altitude [8, 14].

This study employs chorematic modeling, an innovative tool in territorial analysis, to examine changes in quinoa agroecosystems. Chorematic models simplify and visualize complex elements at regional and local scales, providing a unique perspective on spatial dynamics in agriculture [10, 23, 28]. This methodology allows a deeper understanding of the interaction between agricultural practices and the environment, and how this affects the distribution of quinoa and its wild relatives. By applying this approach, our study highlights the importance of chorematic models as decision-making and analytical tools in the context of agroecology and territory management.

### Chronology of crucial moments for quinoa agriculture in Peru

In the highlands of the central Andes between Peru and Bolivia, at 3,800 meters above sea level, lies Lake Titicaca, the cradle of pre-Columbian civilizations and one of the world’s main centers of domestication of plant species grown for agriculture. Physical factors, including altitude and rainfall, strongly constrain the productive capacity of this mountainous area. Altitude determines the limit of animal and plant life; rainfall, changes irregularly and randomly, resulting in alternating years of flooding or extreme drought. Local agricultural societies have existed for thousands of years and have organized themselves around a prosperous cultural heritage strongly linked to this complex environment. In particular, they have developed essential knowledge about their natural resources and launched a process of plant and animal species domestication over the last 8,000 years. Quinoa, along with a whole diversity of related wild species, is one of them. It was domesticated by these populations more than 7000 years ago and then regularly selected by generations of farmers [29]. It has thus become a central element of this region’s agricultural and food production systems. Quinoa and its wild relatives were crucial elements in these Andean societies’ religious rituals and cultural and daily aspects. Pre-Inca indigenous populations practiced agriculture using irrigation, fertilization, rotation, and the construction of terraces to conserve soil fertility, protect crops from frost, and increase agricultural production [29]. The fundamental principles of the socio-economic organization of Andean societies were based on reciprocity, redistribution, and vertical control of the ecology [30]. The Incas (1100–1533 B.C.) consolidated the plot as a subsistence unit in a communal order and taxes. Moreover, they allowed the emergence of private property for the elites. As a result, it was possible to achieve, at the level of the state, magnificent infrastructure work and the accumulation of durable goods, as well as, at the level of the population, the satisfaction of food needs and employment. Both characteristics allow us to appreciate the extent to which the Andean region became an area of significant agricultural and livestock development, which also promoted the dispersion of quinoa throughout their empire [31, 32].

With the arrival of the Spanish in 1535, the organization of the Inca territory was restructured with a substantial impact on agriculture, and thus ultimately on their associated societies. The Spanish regime profoundly modified the Andean administration to impose a system of large properties. At the end of the 16th century, the *hacienda* was established as a unit of agricultural exploitation. This change resulted in a general depression of agriculture, caused, on the one hand, by the irrational economic policy of its first decades. On the other hand, the demographic crisis and the abandonment of the lands broke the comparatively resilient nature-society relationship [22]. Despite the cultural and dietary importance of local plant species, European cereals were imposed on the colonized populations, replacing, among others, quinoa. Nevertheless, even though quinoa cultivation declined sharply until the 1970s, the role of this species in religious ceremonies and local daily life remained considerable, preventing its disappearance [9, 20].

The independence allowed Creole elites to control and own the land in the nineteenth century. However, the newly landed aristocracy retained the traditional character of Spanish crown farming. At the beginning of the 20th century, a productive orientation of agricultural development towards export began. Until the 1950s, the institutional aspects of the current structure were based on a system of occupation and land ownership. The *hacienda* and the local community were the fundamental pillars since they represented the primary forms of access to land and, thus, were the set of social relations that defined the rural society [22, 31].

The year 1970 was chosen as a pivotal year in our historical analysis of the development of the quinoa market in the Andes of Peru. This date corresponds to the beginning of a decade that involves three significant steps in the development of agricultural activity in Peru: the 1969 Agrarian Reform Law, the 1972 General Education Law with the creation of the elementary school in remote areas of the country, and the 1974 Law on the Recognition of Native Peruvian Communities (territorial rights). During this period, Peru was under the mandate of the military government of Juan Velasco Alvarado (1968–1975). The main objectives of this government were: to eliminate the structural causes of social conflicts, redefine the system of wealth redistribution, modernize the economy, and connect the country through the improvement and construction of roads, mainly in the border areas of the country. These reforms gave rise to a liberal industrial and agri-export bourgeoisie. With the Agrarian Reform, most of the large estates (*haciendas*) confiscated from the former owners were transformed into cooperatives. These associations were made up of former *hacienda* workers instead of returning the land to the peasant communities that had been displaced to the poorer lands. Some cooperatives increased production and pooled income more successfully than the previous landowners. However, others could not achieve this, and in the late 1970s, several cooperatives were dissolved. This dissolution divided the cooperatives into individual farms. As a result, two production modalities coexisted from 1970: large productive extensions belonging to the remaining cooperatives and new family-sized farms. Concerning cultivated quinoa, despite the negative connotation of rejection perpetuated since the colonial period, the permanent attachment of the local quinoa-producing populations has allowed it to evolve with the possible natural crosses with its wild relatives, also maintained by local human groups for various uses (food, medicinal, environmental and cultural).

This reality of quinoa began to change in the 1980s, especially during the first regional meeting on plant genetic resources organized in April 1981 by the Andean countries to improve the nutritional situation of the inhabitants by valuing the native plant species of the Andes. Subsequently, various scientific studies have proven the nutritional value of quinoa and other Andean grains and tubers to the point that they have gradually generated worldwide interest in their consumption. Since the 1980s, the main quinoa production areas, located on the shores of Lake Titicaca in Bolivia and the Puno region of Peru as well as on the southern Bolivian Altiplano, have gradually increased until becoming a highly valued export crop for countries in Europe, United States, Canada, and Japan, in search of food with high nutritional value (rich in protein) and certified organic and fair trade [5, 20, 33].

**Fig 1:**
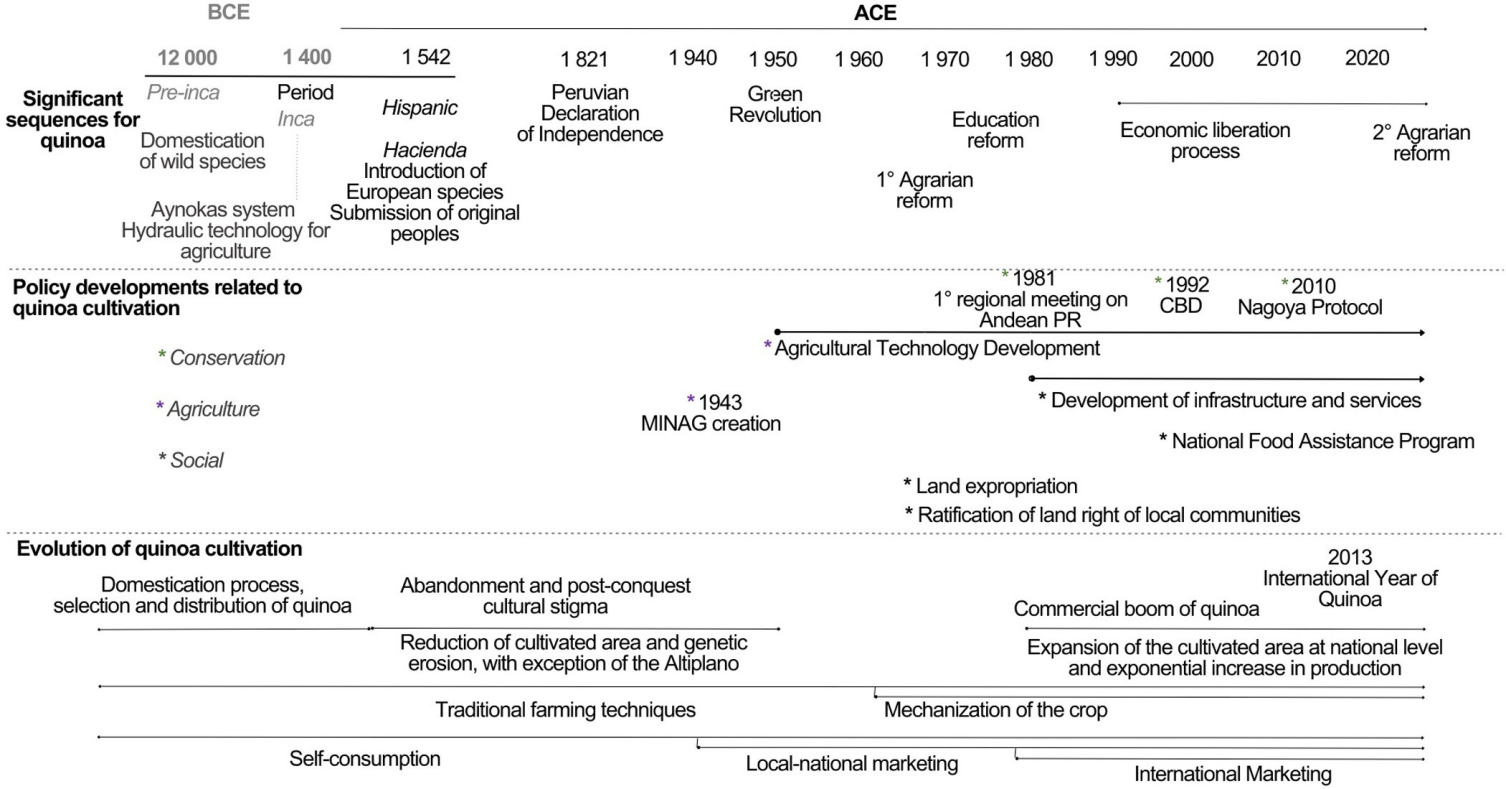
Chronology of the major moments for quinoa cultivation in Peru.

In Peru, the authorization of direct purchase of Andean agricultural products from small farmers in 1994, food aid programs since 2008 that specifically promoted the social inclusion of small local quinoa farmers, and the restructuring of food aid programs in 2012, allowed and promoted the expansion of the area of quinoa cultivated to the Peruvian Altiplano and other regions of the country [5]. The United Nations General Assembly declared 2013 the International Year of Quinoa in recognition of their high nutritional value and the vital biodiversity that the Andean people have maintained [34]. For Peruvians, the quinoa boom and the naming of the International Year of Quinoa have continued to encourage the acceleration of the expansion of agricultural areas in traditional quinoa-growing regions, resulting in competition for land [35]. Studies on the intensification of quinoa production in Peru and Bolivia show impacts on soil degradation and the weakening of the socio-ecological basis of the agrosystem in order to face the multiple challenges of climate change resilience and food security [36–39] (Fig 1).

The significant increase in the cultivated area of quinoa between 2012 et 2015 [39] has transformed both the socio-economic and environmental landscape in the highlands and high Andean zone of the country. References such as Bazile & Baudron [40] and Pinedo et al. [41] detail how production systems have evolved from predominantly subsistence and family farming to monoculture systems with an increasing dependence on external inputs.

This transition has significant implications in terms of environmental and cultural sustainability [39]. The adoption of monoculture practices has raised concerns about biodiversity loss and the consequences of greater dependence on external inputs, such as fertilizers and pesticides, which can have detrimental effects on the environment. Additionally, this shift in production systems represents a move away from traditional agricultural practices, which has profound cultural implications for communities that have historically cultivated quinoa as an integral part of their way of life and cultural identity.

### Study area

**Fig 2:**
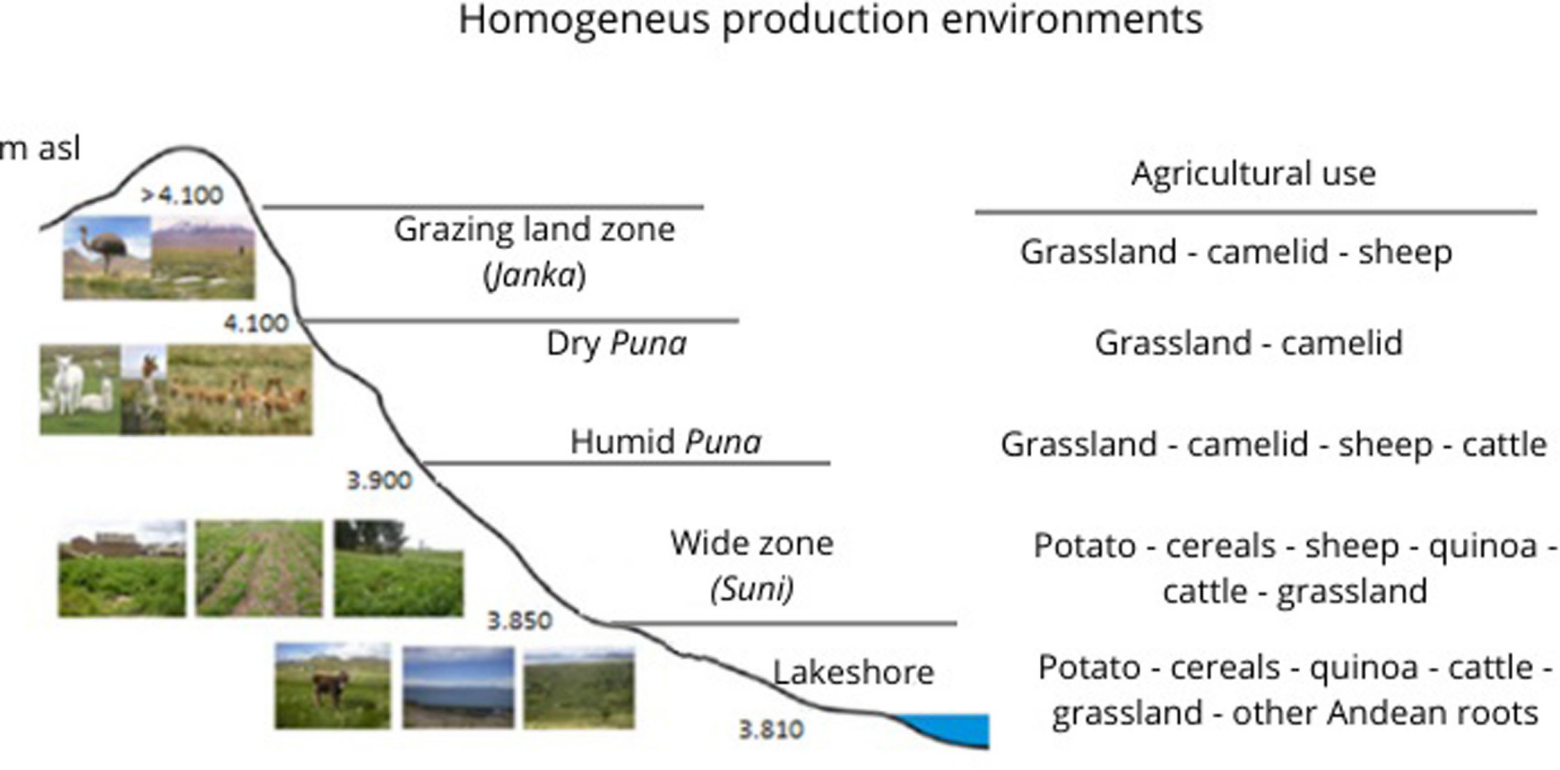
Agroecological zones of the Peruvian Altiplano. Source:[14], adapted from [39].

The work was carried out in the region of Puno on the Peruvian Altiplano in the surroundings of Lake Titicaca. Peru is one of the largest producers of quinoa in the world, and the Puno region corresponds to one of the main areas of quinoa production for export at the national level, with a vital family agriculture based on small-scale production [5]. The study was conducted between 2015 and 2018 under a territorial development approach, integrated and participatory, combining agricultural production and sustainable environmental management, specifically on six sylvo-agro-pastoral communities. Each community has a topographic profile specific to the agroecological zone described in **Fig *2***.

**Fig 3:**
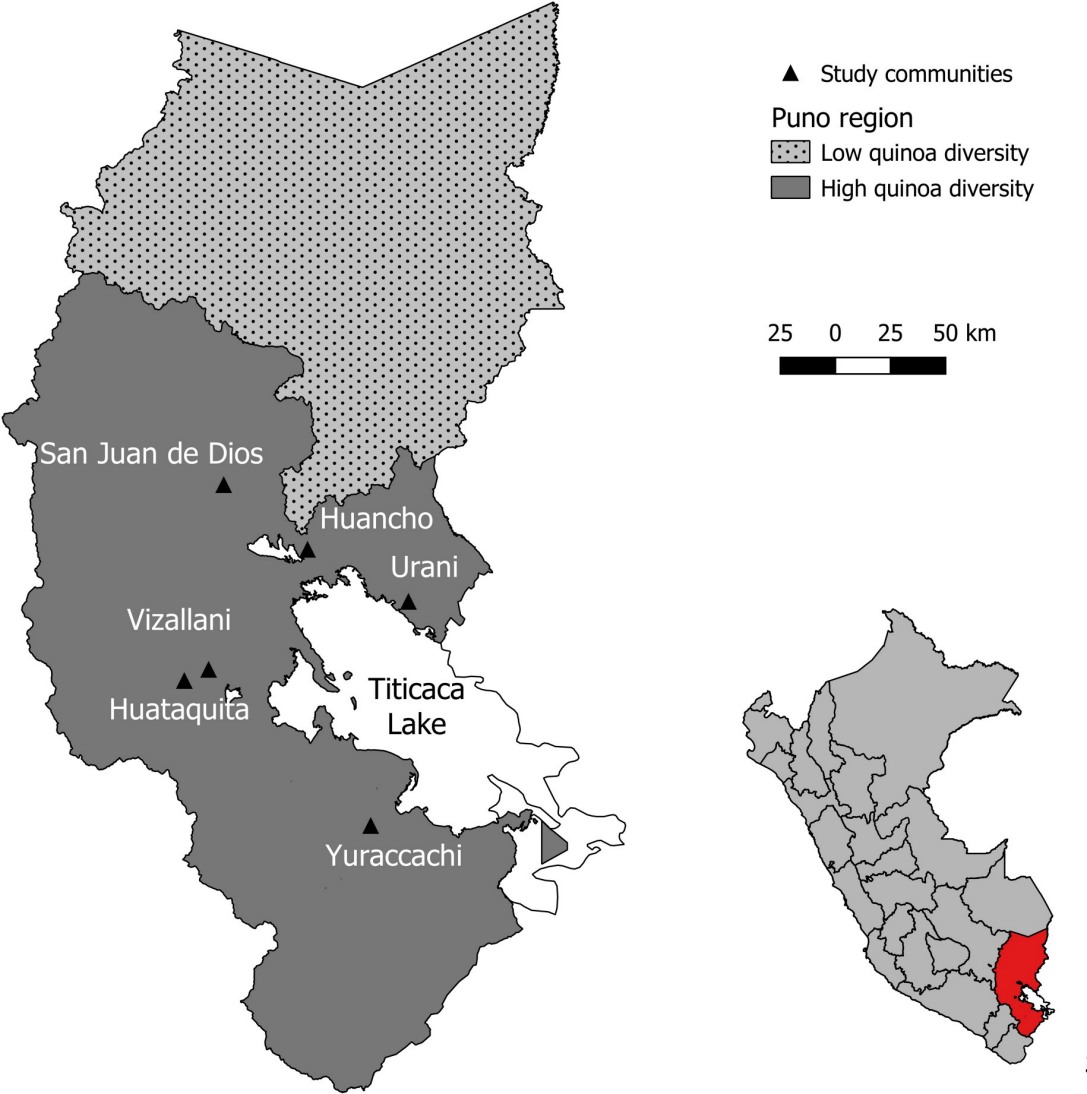
Location of study communities. Source: Reprinted from [14], under a CC BY license, with permission from [GADM database], original copyright [2018–2022].

We were inspired by the characterization of Andean agroecosystems carried out by Morlon, in 1992 and the six communities chosen according to three criteria [14], represent a diversity of agricultural situations [38]. The three criteria of selection are:

- Presence and diversity of cultivated quinoa types: Local researchers have shown that within the Puno region, there is an area considered the center of the greatest diversity of cultivated quinoa (Fig 3) [9]. This diversity of cultivated quinoa is directly related to the diversity of the physical environment.
- North-south gradient: In order to identify a diverse range of quinoa varieties across various biophysical environments, we incorporated the second criterion, which is associated with a north-south gradient reflecting a decrease in the frequency of annual rainfall.
- East-west gradient: the third criterion corresponds to the villages’ altitude, primary factor defining the major regional agroecological zones [39].

The selection of six study sites was done in collaboration with project partners working in public institutions and universities in Peru. They have introduced us to local communities to present our research objectives and ask for their participation. Before starting any field activity, we always received verbal agreements to conduct our research. At each new stage, we presented the communities with the results of the previous stage to validate them and explained our intended next steps, once again seeking their approval. These communities are Urani, Juancho, San Juan de Dios, Villani, Huataquita, and Yuraccocha (Fig 3).

**Fig 4:**
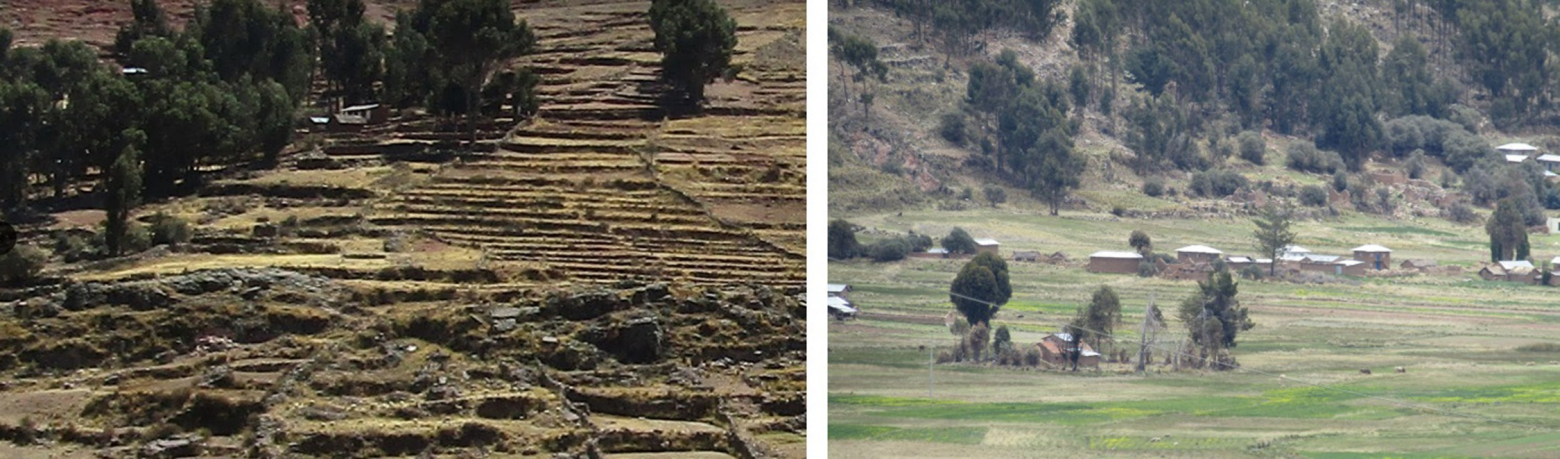
Landscapes of the Andean agroecosystems around the family farming households. On the right a picture of terraced plots in Urani. On the left a picture of plots in the plain of Huancho.

Land use in the family farming systems of the Lake Titicaca basin is characterized by long periods of rest and a strong presence of agroecological practices. In these plots, the management of wild edible plants and crops like quinoa coexist. The fallow plots are also grazing areas for small ruminants. This production system management is developed mainly within a 2 km radius of the farmers’ houses. The agricultural landscape is dominated by three agricultural structures: sloping plots, flat plots, and terraced plots (Fig 4).

## Methodology

### Chorematic modeling

The methodology applied in this research is divided into three key steps:

1. A participatory mapping approach to understand the composition of the agroecosystems related to quinoa cultivation and the presence of these wild relatives
2. The conduct of semi-structured interviews with inhabitants on the trajectory of communities related to quinoa cultivation
3. Chorematic modeling based on the information obtained in the previous steps

#### A participatory mapping process

Workshops were organized with the collaboration of local authorities per community, who were responsible for rallying and convening villagers. The participants per workshop ranged from 14 to 20 people, of which women represented an average of 65%. In order to legitimize the cartographies, we also conducted transects accompanied by several participants in each community. These workshops allowed the construction of maps representing each community’s agrosystem with information on the significant characteristics of the territory and the modes of agricultural production.

#### Semi-structured interviews

Semi-structured interviews with women residents were conducted in an open, conversational setting. A total of 150 individual interviews were applied, with an average of 25 people per community. The population corresponds to a strict parity between ethnic groups, with 75 people from the Aymara ethnic group and 75 from the Quechua ethnic group. The interviews were conducted mainly in Spanish, but in some cases, we were accompanied by a translator in the local language. The people who voluntarily participated in the interviews were mainly adults over 50. The details of the interviewed population can be found in Table *1*. The analysis of the interviews was carried out according to the method proposed by [42]

**Table 1:** Structure of the interview population in the six villages.

Ethnic group	Women			Men			Total
	≤ 50 years	>50 years	Subtotal	≤ 50 years	>50 years	Subtotal	
**Aymara**	14	24	38	11	26	37	75
**Quechua**	23	30	53	3	19	22	75
**Total**	37	54	89	14	45	61	150

#### Chorematic modeling

**Fig 5:**
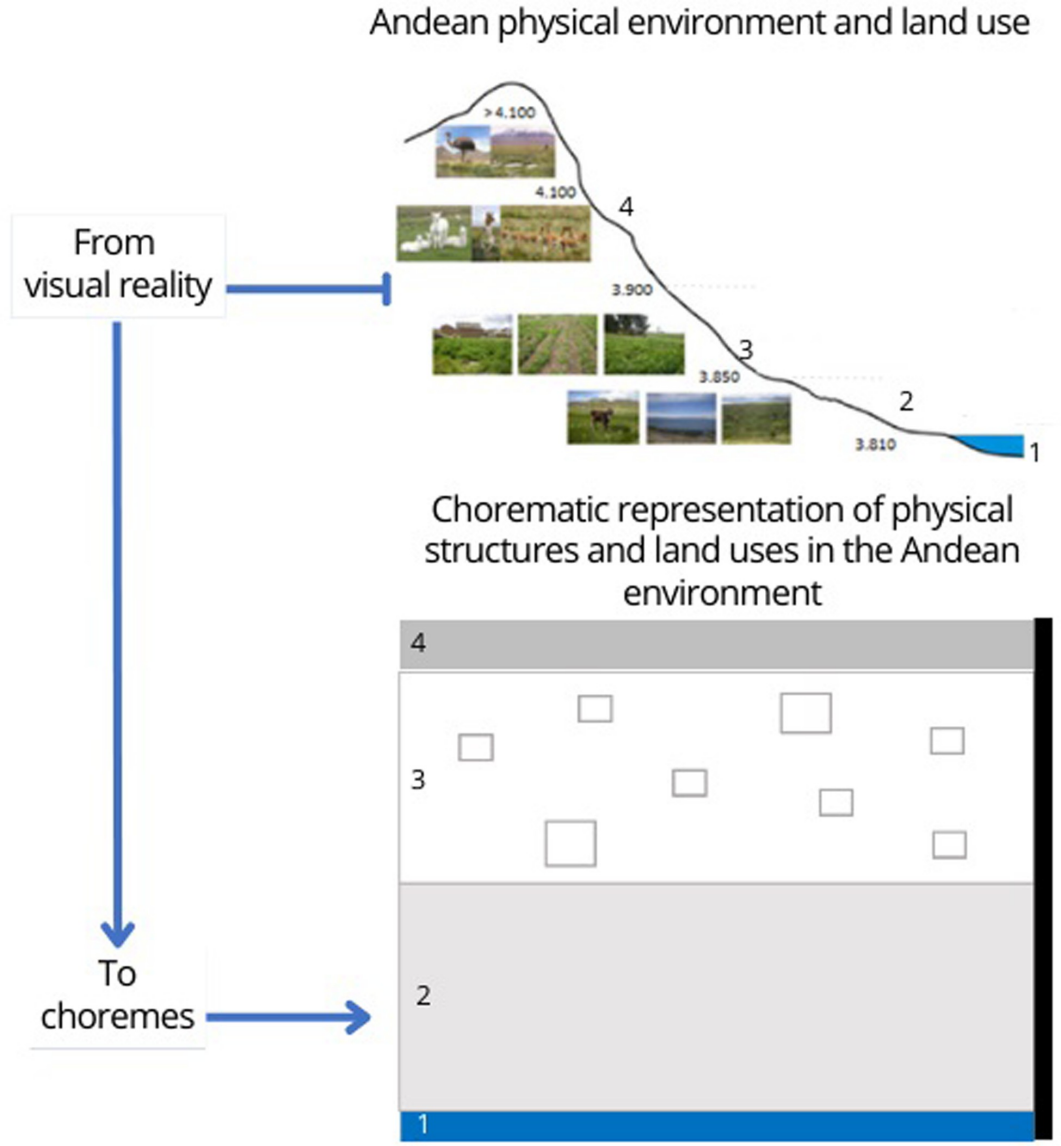
Structure of Andean agroecosystem in ecological levels. Explanatory correspondence between, on top, the illustration of the physical environment, with photographs of the landscapes according to the altitude (major factor of differentiation of the ecosystems of the Andes), and underneath, the choremes.

The last step is the passage from participatory drawing to chorematic modeling. Chorematic modeling is a tool for representing reality, forecasting, and studying the territory’s spatial dynamics [24]. Therefore, this type of graphic representation is a platform for consultation of territorial actors and a powerful tool for debate in terms of decision-making for territorial development. The method of chorematic modeling applied in this work is inspired by Brunet’s the seven structures and four basic elements [24]. The 28 choremes are synthesized into a mesh, grid, attraction, contact, tropism, territorial dynamics, and hierarchy. For our article, the application of the chorematic representation of the communities studied allowed us to highlight the essential elements of the particular dynamics at work over the last 50 years, due in particular to the growing interest in quinoa by the international markets. Knowing that until now, no classical representation mode has allowed us to update this dynamic and its determinants. The first step in the processing of data on the territory was to identify the structure of the Andean agroecosystem in 4 ecological levels: from the lake to the mountain range (Fig 5)

The next step represents an epistemological leap since it models the agroecosystem, i.e., deduces the shape of the distribution of the agroecosystem’s elements from its location in time and space. The geometric form representing each element no longer has a value of generalization, in the cartographic sense, of the information but a logical value. Then the figures adopt a standard and simplified graphic language. Also, the model has neither scale, orientation, nor dating, contrary to the map and the diagram.

For the chorematic work, first of all, we identified the essential elements to remember and the central relationships involved to facilitate the dialogue about the evolution of the landscape concerning the boom of the cultivated quinoa in Puno. Currently, the six villages studied are at different times about the development of the agricultural sector and quinoa, with the year 1970 being pivotal for the development of agriculture in Peru (we explain this in the next paragraph). We modeled their socio-spatial dynamics in two stages:

▪ The first is the "pre-1970" period, for which the model represents an ordinary reality for all villages;
▪ A second contemporary period, "after 1970", represents the reality of three villages most involved in the quinoa market today (San Juan de Dios, Huataquita, and Vizallani). The model related to this period corresponds to a possible scenario shortly for the other villages studied (Huancho, Urani and Yuraccachi) that are in an initial phase of commercial production of quinoa.

## Result

For the chorematic modeling before and after 1970, the consideration of temporality, with possible changes over time, in the interviews with the 150 people was helpful.

### Physical structure and different land uses: Use of the Andean environment by the six villages studied before and after 1970

**Fig 6:**
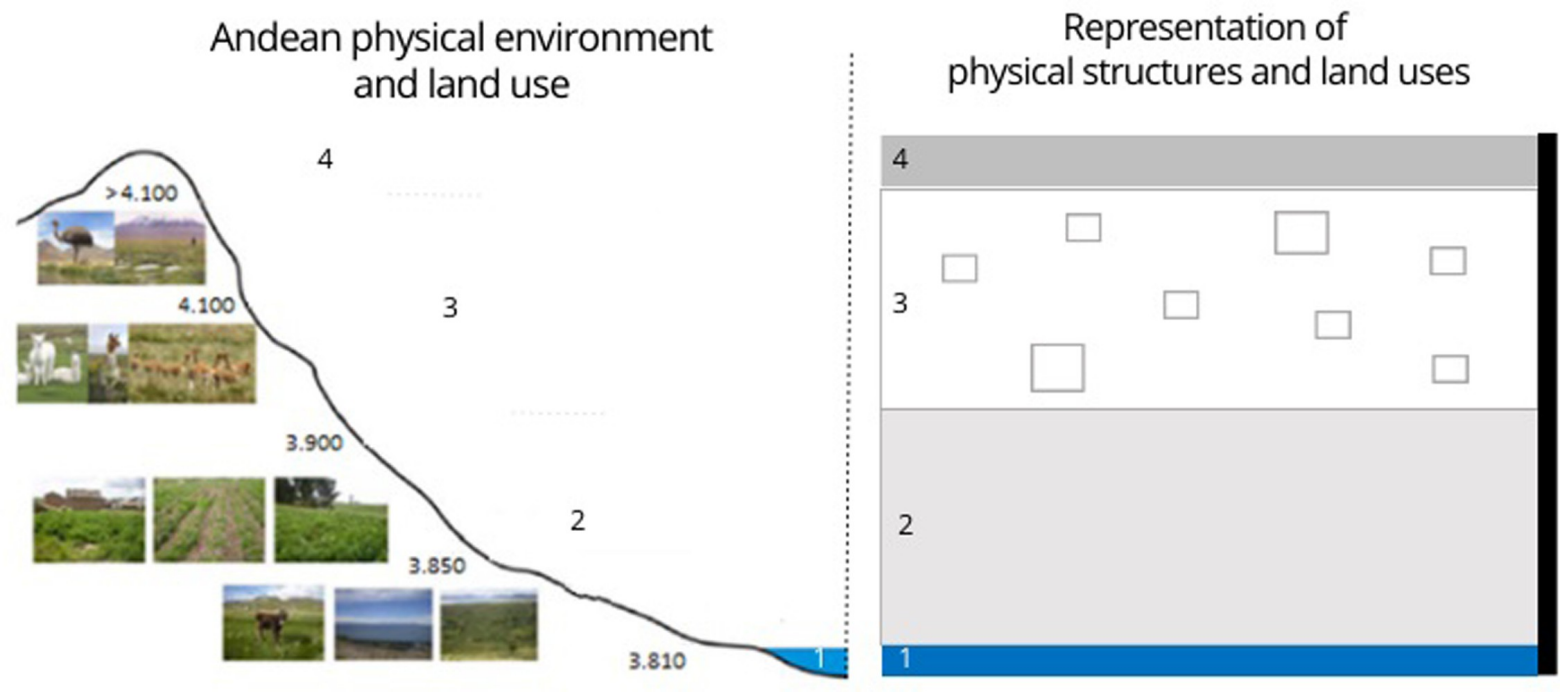
Chorematic physical representation characterizing the six study villages. Explanatory correspondence between, on the left, the illustration of the physical environment, with photographs of the landscapes according to altitude (major factor of differentiation of the Andean ecosystems), and on the right, the choreme (here for before 1970), with the physical elements retained here: (1) Water source ‐ (2) Pasture ‐ (3) Quinoa cultivation, other crops and fallow ‐ (4) Cordillera. The colored vertical band, from light to dark, represents the altitudinal gradient, from the lake level to the highest peak.

The first choreme developed is a physical representation characterizing the six villages. Fig 6 shows the correspondence between the environment in its visual reality (photographs) and the chorematic representation. Land uses (quinoa and/or other crops, fallow land, plantations, and others) are added to show how the evolution of quinoa cultivation in the international market modifies socio-spatial dynamics. In the introduction to the methodology, we explained the evolution of the last decades in Peru, with the year 1970 as a turning point. The forms of a social organization influence the territorial organization of the villages.

#### Use of the Andean environment by the six villages studied before 1970

**Fig 7:**
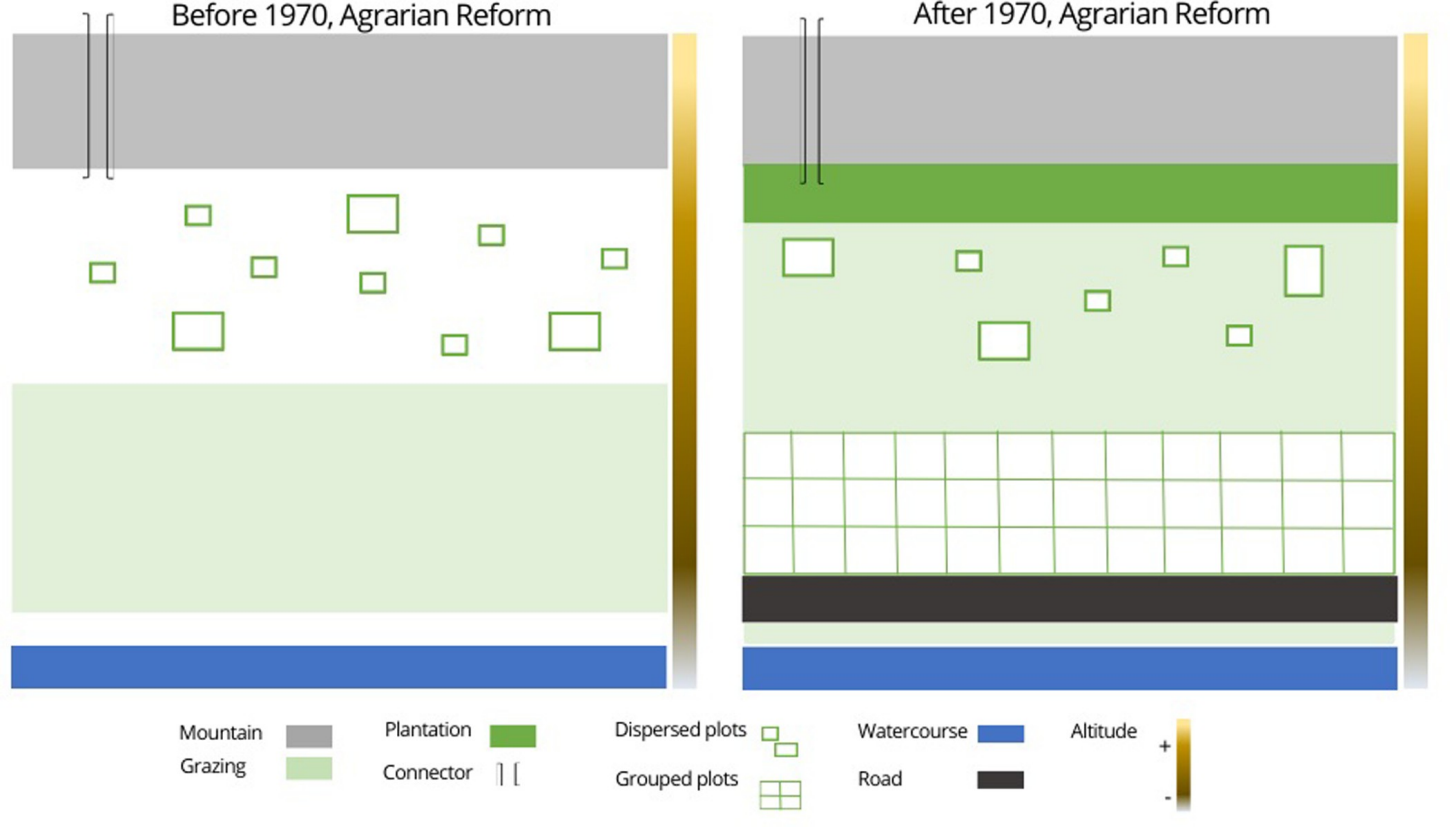
Physical structures, and different land uses before and after 1970. Chorematic representation of the physical structures of the different land uses of the Andean environment: top, before 1970; bottom, after 1970. The colored vertical band, from light to dark, represents the altitudinal gradient, from the level of Lake Titicaca to the highest peak.

Before 1970 (Fig 7), the territorial organization of villages reflected the planning of family and communal economic production in the village. The village’s lowest part was the primary source of water for the village and the animals. The plains corresponded to areas of collective use for grazing. The villagers have continued to use the traditional technique of in-ground farming. This technique minimizes the risk of frost, provides greater exposure to the sun, controls water drainage, and allows for better aeration of the agricultural soil. The scattered plots show the use of a diversity of soils and micro-topography, which allows diversified management of crop varieties to secure production and manage risks. Crop rotation is strongly linked to the ecological characteristics of each plot, which allows for the maintenance of agroecosystem variability, ensuring long-term food security. Thus, the system allowed soils to recover during long fallow periods. These fallow lands were also used as communal pastures. At that time, the system was maintained by a dynamic integration between livestock and agriculture. The Cordillera represents the highest ecological level of the agroecosystem. It was a space of passage, a connector of the villages with their neighbors to exchange products, and a meeting place for the shepherds during transhumance.

In this context, the Cordillera represents not just the highest ecological tier of the agroecosystem [3], but also plays a vital role in biodiversity management and agricultural sustainability. Studies conducted in the Andean Cordillera illustrate that these mountain systems have historically been central to the formation of diverse and adaptive agricultural practices. For instance, land use systems in Colombia’s Eastern Cordillera reflect a complex interplay between biophysical conditions and human use, including agriculture and livestock [43]. Additionally, the climatic variability and altitudinal gradients in the Andes Cordillera have been fundamental in developing resilient agricultural practices and conserving genetic resources, as detailed in research on observed and predicted climate changes in the Andes Cordillera [44]. These characteristics render the ’Cordillera’ a unique agroecosystem, marked by its biodiversity, adaptability, and cultural significance.

#### Use of the Andean environment by the six villages studied after 1970

After 1970 (Fig 7), government investment in road connectivity improved the integration of remote villages. The construction of roads in or near villages translates into access to public transport for villagers and potential opportunities for acquiring of motorized travel for the family. The most appropriate location for road construction is parallel to watercourses. Currently, the agricultural use of terraces is no longer a common practice. This practice can only be observed in some areas of the agroecosystem, mainly in the lower parts of the slopes where there are still permanent or temporary crops for self-consumption. With the development of the quinoa market, farmers will move the cultivated area from the slopes to the plains. This strategy will also group the plots to have a larger production area. Eucalyptus (*Eucalyptus globulus*), originally from Australia, has become a significant component of the Peruvian Andean ecosystem, mainly due to an Agrarian Reform program, and its ability to grow in this environment [40]. The program was intended to provide the peasants with a source of fuel, building materials and perhaps a commodity. The villagers have continued with projects to plant these trees to avoid landslides affecting the Altiplano during heavy or frequent rains. These trees also create a microclimate.

### Chorematic of socio-territorial dynamics at work before and after 1970

#### Socio-territorial dynamics at work before 1970

**Fig 8:**
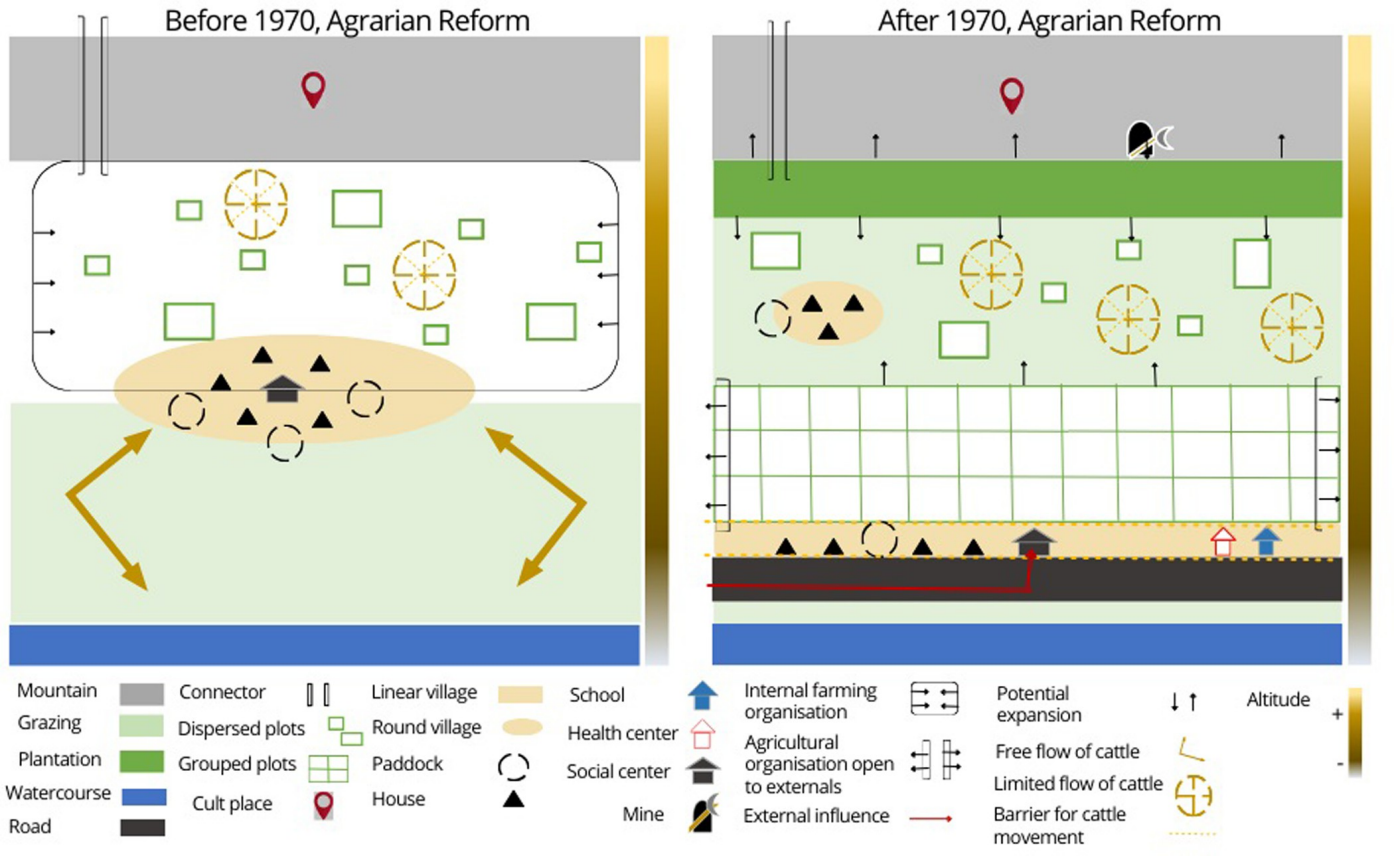
Chorematic representation of the socio-territorial dynamics of the Andean environment. Top, before 1970; bottom, after 1970. The colored vertical band, from light to dark, represents the altitudinal gradient, from the level of Lake Titicaca to the highest peak.

Before 1970 (Fig 8), the old land use and crop rotation system allowed for the distribution of community resources through norm-setting and community decision-making. A dynamic interdependence between crops and livestock supported this agropastoral production system. This dynamic interdependence means that the level of productivity of one of these components directly affects the level of productivity of the other. The production system was based on the traditional cultivation of several varieties of quinoa, tubers, cereals, and fodder for livestock. The inhabitants also raised various local breeds of animals. The grazing area on the plains, away from the crops, was strategic to allow free movement of livestock and easy access to the water supply for the animals. On the Cordillera, in the highest parts, is the space of ritual worship and social meeting with the outside.

Fig 8 is a chorematic representation of the socio-territorial dynamics superimposed on the physical structures and land uses in the Andean environment before and after 1970.

#### Socio-territorial dynamics at work after 1970

**Fig 9:**
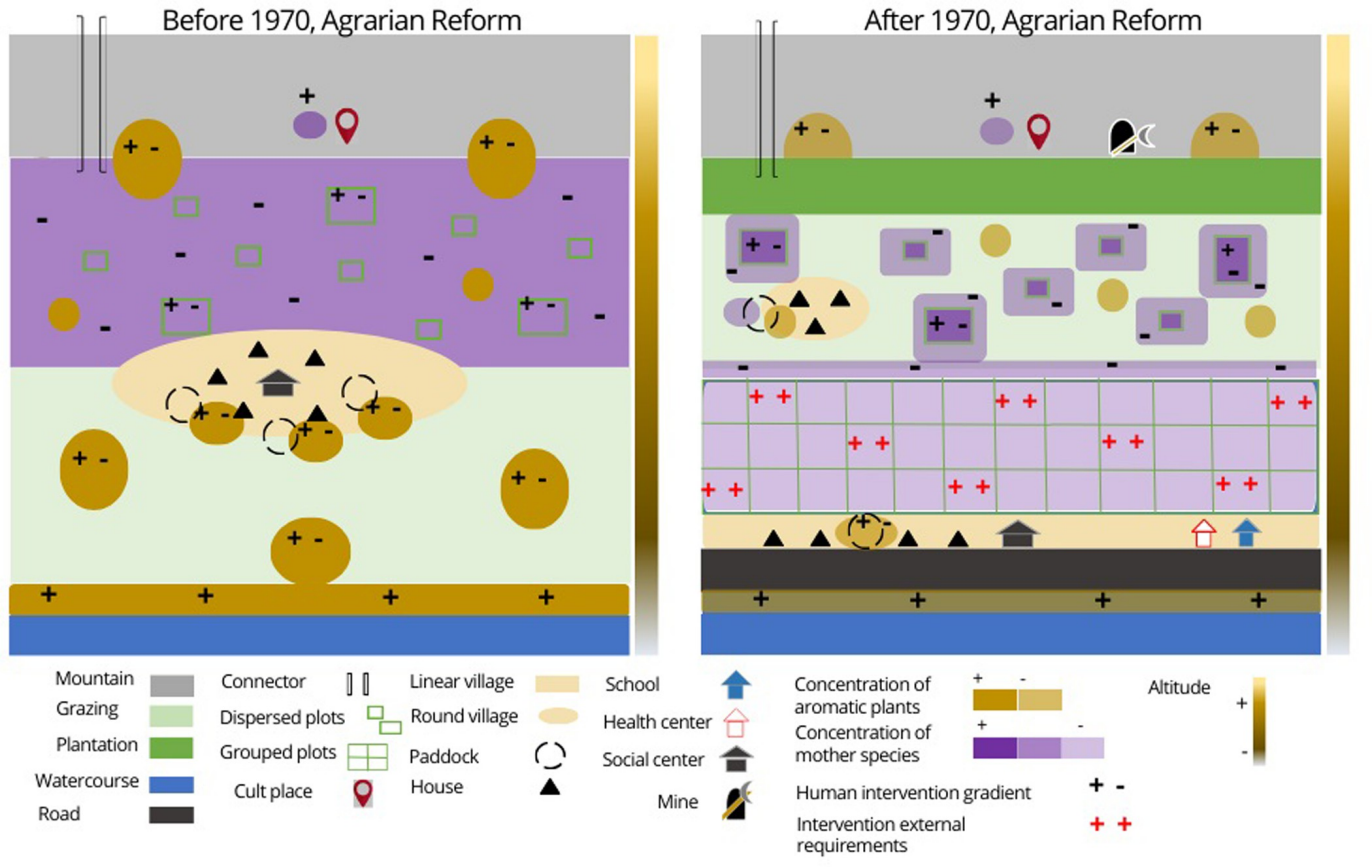
Chorematic representation of the presence of wild relatives of cultivated quinoa in relation to socio-territorial dynamics. Top, pre-1970; bottom, post-1970. The colored vertical band, from light to dark, represents the altitudinal gradient from lake level to the highest peak.

After 1970 (Fig 9), villagers added tree plantations to the agropastoral system. Two of the six villages (Urani and Juancho) have tree nurseries. The plantations of exotic species, such as eucalyptus, have helped to regulate soil infiltration conditions and storage capacity during the rainy season, in addition to producing wood and economic surplus. On the other hand, planting decreases grazing areas, especially with the gradual increase of this activity towards higher areas, and the plains. Expanding of tree plantations in low areas is associated with creating more sheltered spaces. The trees create microclimates that protect the crops from low temperatures. The construction of the road also becomes an axis of change in spatial organization. The houses were once semi-concentrated between the agricultural space and the grazing area. The social meeting place was an open space, usually in the shade, with enough space to receive all the inhabitants and deal with issues of general interest. Currently, houses are constructed at the road’s edge, following a linear morphology. The road and access to motorized mobilization make it easier for villagers to participate in events such as fairs to sell or trade their products. The use of the mountain range as a straight connector remains, but the number of people using it is decreasing.

Today, various factors have led villagers to reduce their livestock numbers. One of these factors is the barriers to livestock movement, such as the road, the linear village, and the clustering of farm plots together. Other factors have also contributed to the decline in livestock numbers, such as increased work time in agricultural activity to meet market demands, reduced grazing land in competition with agricultural land, and the search for economic income outside the village to improve the quality of family life. The social meeting center is now a closed building. In the new meeting center, only a part of the population usually meets to discuss topics of specific interest. If the topic to be discussed is of general interest (such as a ritual or celebration), the meeting is done in an open place. This closed building can also be the village school. The schools were built during the military government as a strategy to integrate the various ethnicities through access to education and learning Spanish.

In terms of agricultural organization, the villagers form two groups: those who are cooperative members and those who are not—those who are not interested in the cooperative work the land independently. Farmers who have become cooperative members must work with other villages belonging to the same cooperative. They also work with outside institutions involved in the quinoa production chain. Villagers not interested in the agricultural market have sought other economic opportunities. The most important mining activity in the Puno region is located in Sandia, Lampa, Carabaya, and Melgar (of the four provinces mentioned, Melgar is the only province with villages studied). The mining exploitation is mainly gold, copper, silver, and clay. Several men from each village move to these provinces to work in the mines, leaving the cattle and agricultural work to their families. However, as the Cordillera is rich in minerals all over Puno, in some villages (which we will not name to respect the demand of each village), men (the beliefs of the villagers do not allow women to work in a mine) have begun to develop a clandestine mining activity.

### Chorematic representing the relationships between socio-territorial dynamics and distribution of wild relatives of cultivated quinoa

Fig 9 is the chorematic representation that juxtaposes the presence of wild relative species of cultivated quinoa concerning socio-territorial dynamics **before and after 1970** to the previous ones. The seven species of wild relatives of cultivated quinoa are present in various parts of the Andean agroecosystem. For *C*. *quinoa* Willd. a group of three wild relatives is recognized as direct parent species. Interviewees’ perception of this group as the parent species of quinoa is based on three simultaneous criteria: the morphological similarity of these plants to quinoa, their presence in the cultivated quinoa plot, and their ease of crossing with quinoa. The interviewees recognize the other four wild relatives species as aromatic plants [18].

#### Relationships between socio-territorial dynamics and distribution of wild relatives of cultivated quinoa before 1970

Thanks to the ritual and social activities of the villagers, the mother species grow at high altitudes, associated with places of worship. Thanks to human intervention, these mother species remain in these spaces until today. The new generations reappropriate these Andean rituals in which the mother species continue to be included. When quinoa is cultivated for self-consumption, the selection of plots and varieties meets very different criteria than those used for commercial cultivation. Quinoa is ideally grown in rotation after potatoes on frost-free slopes. On the other hand, since *cañihua* (*C*. *pallidicaule* Aellen cultivated) is more resistant to frost, it is grown on soils more exposed to a cold microclimate and in rotation after bitter potatoes. In the traditional conception, the quinoa cultivation does not require specific preparation of the land for sowing. Being in rotation after potatoes allows the soil to be sufficiently loose, and the residual organic fertilizer from the previous crop will meet its growing needs. Farmers will plant various quinoa varieties in different plots and within the same *chacra* (In Quechua, "Chakra" means an agricultural or farming field). Although some *chacras* in the traditional method were plowed with oxen, others were not. The small size of the terraced *chacras*, the rocky characteristics, and the problematic access means that, in this case, the land is prepared by hand with beaks (pointed tools). Leaving grass inside the *chacra* is considered a nutritional resource for the soil. At the same time, a percentage of parent species within the *chacra* is considered favorable to ensure that at least these plants can be harvested in case the cultivated quinoa crop is lost. The degree of human intervention in terms of the presence of the mother species in and around the cultivated plots has allowed for the formation of a higher concentration and natural crossbreeding over the past (before 1970).

Training received from institutions such as CIRNMA (Natural Resources and Environment Research Center), INIA (National Institute of Agricultural Research or Agrarian Agencies recommends the use of modern techniques to increase agricultural production and productivity. The villages studied used chemical fertilizers containing nitrogen and phosphorus. However, these fertilizers are now almost absent and no longer recommended because they increase the cost of production and because they cause environmental pollution problems. For organic quinoa production, natural manure comes from the livestock the villagers raise and the compost they have learned to make. The weeding and thinning of the crops are done rigorously to avoid competition for nutrients between plants as much as possible, preventing the spread of quinoa’s parent species. These new practices do not prevent the mother species from developing in and around the cultivated plot, but their concentration decreases.

Farmers outside the cooperatives who do not participate in institutional training still imitate the new practices. The use of improved varieties (some more resistant to pests or diseases) is aiding the harvest. The greater dispersion of cultivated plots currently hinders the pollination of quinoa’s parent species. Although some farmers follow traditional methods, the increased distance between plots decreases in the concentration of parent species. So-called aromatic species of wild relatives of cultivated quinoa are usually associated with livestock traffic. Villagers used to take their livestock to graze near waterholes, where vegetation and especially these species, are more abundant. These species grow in clusters on the plains and serve as food for cattle. Although they are not the preferred food of the animals, they become food items in times of grass shortage. The dung that naturally remains on the ground facilitates their development. It is common to find them also in pens and around houses, in humid and shady places.

The fallow lands were also collective pastures. The dynamics between livestock and agriculture bring these species into the agricultural area, but with low concentration. The presence of the species in the cultivated plot is still considered positive by the farmers. Their presence nourishes the soil, and the fact that they are bitter plants helps to control pests. These species grow at high altitudes, being present in the hills and isolated from the human population. In general, it is the shepherds who know where they are. These species, which grow in ravines and where the soil is soft, are collected for medicinal purposes when needed.

#### Relationships between socio-territorial dynamics and distribution of wild relatives of cultivated quinoa after 1970

After 1970, with new household products, new chemicals for agriculture, and the development of mining activity in the villages, the growth and use of these species became more limited. Roads and stream pollution are reducing the presence of aquatic and streamside biodiversity. Chemicals and new agricultural practices allow less growth of these species in cultivated plots. Water containing domestic products such as shampoos or commercial detergents is discharged near homes, limiting the development and concentration of these species. The plants that continue to grow in these areas are no longer used by villagers, as they say that household products can interfere with the medicinal and nutritional benefits. A health center in or near the village allows them to access public health treatments. With road connectivity and transportation, villagers can travel to hospitals and pharmacies in town. Not using these species frequently decreases villagers’ perception of their presence and knowledge of where they can find them.

The acidification of the soil by the new eucalyptus plantations does not allow the growth of these aromatic plants, which results in a cessation of their presence between the slopes and the tops of the hills. Local populations have already perceived the environmental damage caused by exotic tree plantations. In order to remedy these effects, environmental institutions have implemented reforestation projects with species native to the Altiplano.

Today, practices often deviate from the traditional method. The pressure to increase quinoa production to increase family economic income has resulted in an increased demand for land for cultivation. As a result, resting times have been reduced or eliminated for some lands. Farmers explained that large plots on low slopes and plains are plowed and seeded with oxen or farm machinery. In addition, these spaces allow the trenches for sowing quinoa to be set with greater separation between them. Currently, with the shift of the agricultural production area from the slopes to the plains, soil preparation and sowing is becoming more critical. The advice to increase crop productivity leads to fertilizing the soil and maintaining a low population of weeds. In addition, it is considered that the quality of the seed will guarantee the best productivity of the crop. For farmers, it is important to obtain registered or certified seed that maintains the essential characteristics and purity of the variety. Another selection criterion is the size of the grains. A large quinoa grain represents a more significant amount of nutritional reserves, capable of tolerating adversities during the early phenological stages of the plant.

## Discussion

### Choremes highlight qualitative, temporal, and socio-spatial changes

Based on a historical and systematic approach, we presented how the dynamics of agroecosystem development according to the production system of *C*. *quinoa* Willd. in the Puno region imposes a change in the territory with the emergence of new agricultural practices. In the six villages studied, through the choremes, we analyzed the historical process of intensification of quinoa-based cropping systems and how these recent production patterns take or do not consider the spatial distribution of wild relatives of cultivated quinoa.

Our results show the beginning of a shift away from old agricultural practices and the gradual transition to new market-oriented practices that need to be included in a reasonable time frame for farmers to adapt and incorporate them into their cosmovision and integrated spatial design. Although indigenous quinoa varieties have commercial value, more and more farmers are choosing to plant certain varieties with more homogenous production (this homogenization is aimed at standardizing export post-harvest operations) to provide income for their families. On the other hand, local authorities, some of whom are mobilized and proactive, participate in development projects since they are the first to decide whether or not the project is suitable for their community ‐ this is the case, for example, in San Juan de Dios and Vizallani, where the president of the quinoa cooperative was also elected as the village president. In this context, where the development of agricultural production by traditional methods in the ancestral logic seems to be blocked due to the lack of human resources to achieve the desired production volumes, it is through the mobilization of other factors of production, mainly structural or technical, that agricultural development is now taking place.

The gradual commercialization of quinoa in the Andes has accelerated the widespread implementation of new mechanized cropping systems in the lowlands resulting in natural resource degradation. In her analysis of the specific case of the southern Bolivian Altiplano, Vieira Pak’s [15] dissertation identified three factors of change in quinoa cultivation that triggered cascading environmental problems:"(i) structural changes within the production system with the advent of mechanization; (ii) widespread expansion of quinoa cultivation in the plains, to the point of saturation of arable land; and (iii) agrarian crisis of the system." Two problems were particularly highlighted for Bolivia [15]. On the one hand, the new cultivation practices are not adapted to the ecological and social sustainability of the agricultural systems, given the fragile agro-ecological conditions of the production areas, which are under pressure for their governance and, on the other hand, the organic certification standards do not directly assure the sustainability of quinoa production in place.

In the image of what many researchers have observed in the Andes [3, 30, 41], the social organization and the circulation in and out of the agroecosystem satisfy the imperatives of the spatial distribution of the activities (agricultural, mining, breeding, and others) in the different zones of the territory [3]. In addition, the specialization in agriculture and the production of local varieties adapted to each agroecological stage, as well as the mutual aid that characterizes the Andean social system, have made it possible to find a diversity of activities distributed in different places for the exchange of seeds or other products. Unlike Murra’s model [30], today, the complementarity of the resources that families need to live is no longer achieved through managing different cropping systems distributed over several agroecological levels. We have observed that today families are combining agriculture with urban activity, and any work in addition to agriculture is accompanied by a double residence in the city. Urban employment is a complementary resource and a quest for new opportunities to guarantee a better quality of life for the new generations since it allows children to attend secondary or even higher education.

Our deliberately simplified representation of Andean agroecosystems needs to consider all the elements that makeup socio-ecosystems. We have isolated the elements related to historical processes and dynamics of change related to quinoa cultivation to compare two time periods (before and after 1970). The elements chosen by our method follow a logical value that allows us to understand the evolution of agroecosystems at the local level with influences at a national scale. In our chorematic model, the specificities of each village are deliberately absent (although there are shifts between villages).

## Conclusions

The results of this study reinforce the need to think about attractive technological alternatives for these farmers in the context of quinoa cultivation, which is not only aimed at the market but is strategic for their family food security.

However, agricultural technology development has focused mainly on productivity as the overriding evaluation criterion and, to a large extent, on experimental fields that only consider some of the elements of local farmers’ agroecosystems. Of course, farmers also want to increase the productivity of their crops. However, the vision guiding technological development must also consider the particularities to which these agrarian societies are subject. The presence and management of wild relatives of cultivated species represent a double challenge for Andean family agriculture, on the one hand, to improve productivity and, on the other, to preserve food and nutritional security in the face of the effects of climate change.

In light of the evolution of quinoa cultivation and the management of its wild relatives, Peru’s agricultural policies highlight the precariousness of Andean family agriculture. However, they have been much less effective concerning social inequalities and the conservation of wild germplasm as an element of resilience for farmers. On the other hand, even though they remain marginal, local farmers, with their traditional practices, contribute to genetic diversity and food security.

The perspectives opened up by our research concern the sustainability of management practices and agricultural practices with the aim of dynamic *in situ* conservation of wild and cultivated biodiversity. A historical perspective of the results, made possible by the choremes, allowed us to question the evolution of the local communities’ management practices of these different species. In terms of involvement, two types of projects could be considered. The development of projects that consider the maintenance of the presence of wild relatives of quinoa in the cultivated field is favorable to the introduction of genes of interest to help quinoa adapt to changing ecological conditions under the effects of climate change. Also, specific projects of *in situ* conservation of agro-biodiversity, which consider the natural and cultivated areas as a coherent whole, represent a way to manage gene pools important for agriculture and world food in order to have the plant material (phytogenetic resources) necessary for the future of the varietal improvement of Andean species.

However, the projects to be developed must go beyond the disciplinary boundaries between the sphere of hard sciences and human sciences. A socio-ecosystems approach could improve the articulation of these two spheres of knowledge.
